# Supplementary material for: Inactivation of bacteria using synergistic hydrogen peroxide with split-dose nanosecond pulsed electric field exposures
Source: PLoS One. 2024 Nov 18;19(11):e0311232. doi: 10.1371/journal.pone.0311232 (PMC11573215; doi:10.1371/journal.pone.0311232)
Supplement: S1 Fig — (PDF) [file pone.0311232.s001.pdf]

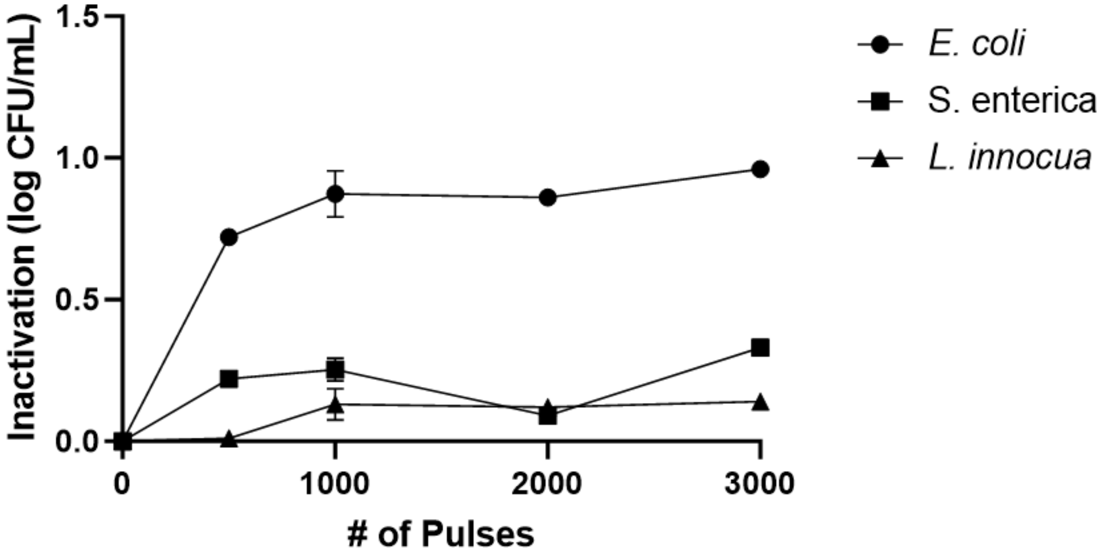

Figure S1. Log reductions of *E. coli*, *L. innocua*, and *S. enterica* for PEF with a single train of varying numbers of 600 ns pulses at 21 kV/cm in the absence of H_2O_2. Each data point represents a single replicate, the 1000P sequence is represented as the average of triplicate analysis with error bars representing standard deviation.
