# Supplementary material for: Inactivation of bacteria using synergistic hydrogen peroxide with split-dose nanosecond pulsed electric field exposures
Source: PLoS One. 2024 Nov 18;19(11):e0311232. doi: 10.1371/journal.pone.0311232 (PMC11573215; doi:10.1371/journal.pone.0311232)
Supplement: S2 Fig — (PDF) [file pone.0311232.s002.pdf]

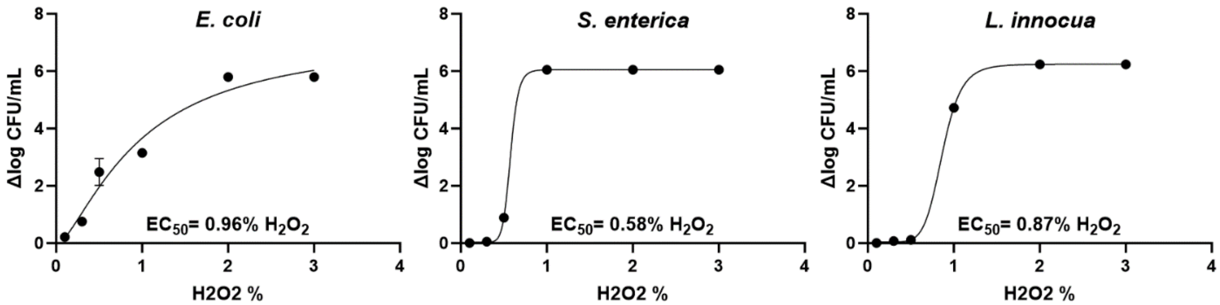

Figure S2. Log reductions of *E. coli*, *L. innocua*, and *S. enterica* for varying concentrations of H_2O_2. Each data point represents the average of triplicate analysis, error bars represent standard deviation.
