# Supplementary material for: Inactivation of bacteria using synergistic hydrogen peroxide with split-dose nanosecond pulsed electric field exposures
Source: PLoS One. 2024 Nov 18;19(11):e0311232. doi: 10.1371/journal.pone.0311232 (PMC11573215; doi:10.1371/journal.pone.0311232)
Supplement: S3 Fig — (PDF) [file pone.0311232.s003.pdf]

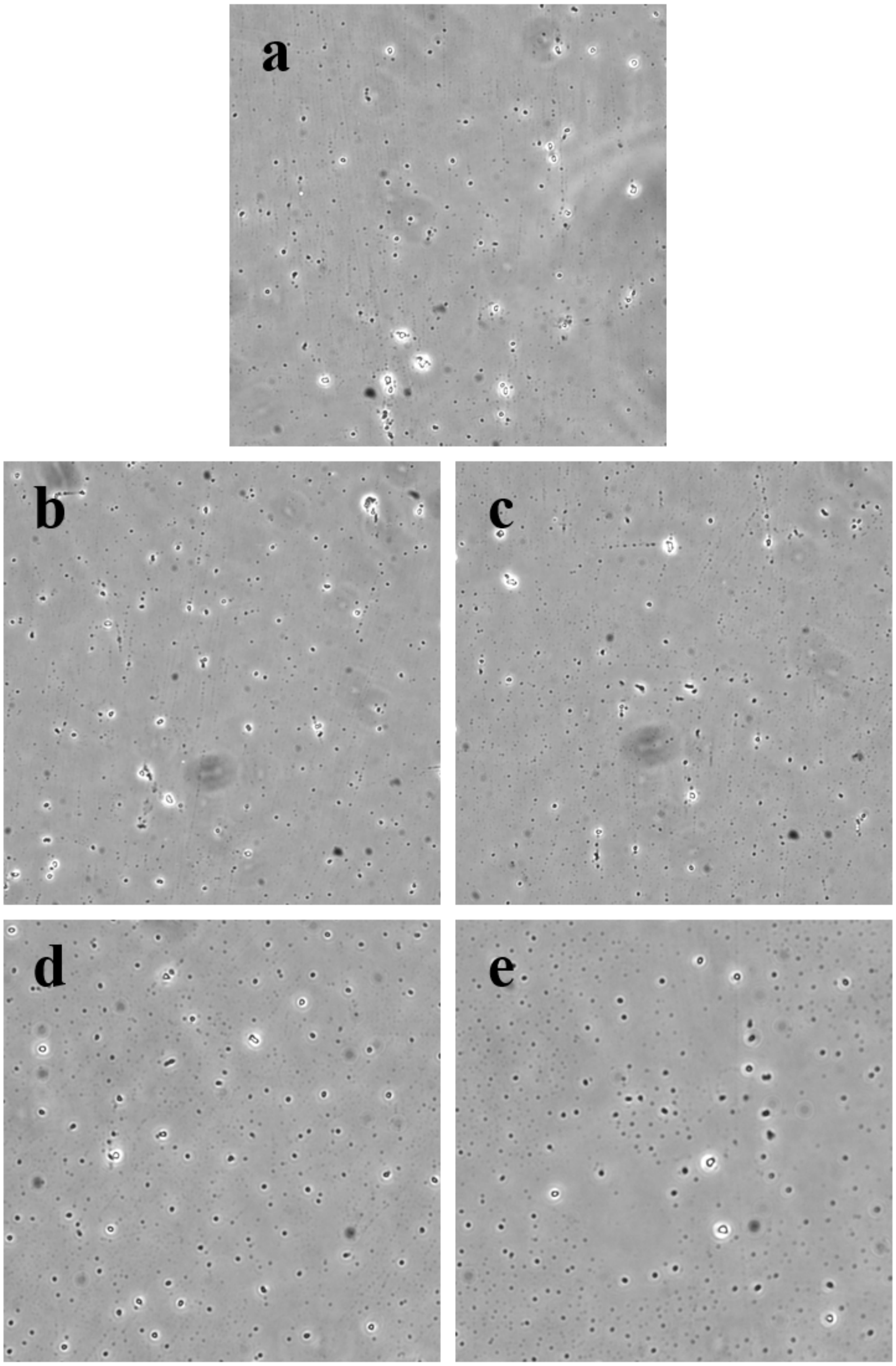

Figure S3. *L. innocua* suspended in Gomori buffer with no treatment (a), in 0.1% H_2O_2 after 500 pulses and a 5 min contact time (b), in 0.1% H_2O_2 after 500 pulses and a 30 min contact time (c), in 0.3% H_2O_2 after 500 pulses and a 5 min contact time (d), and in 0.3% H_2O_2 after 500 pulses and a 30 min contact time (e).
