# Supplementary material for: Inactivation of bacteria using synergistic hydrogen peroxide with split-dose nanosecond pulsed electric field exposures
Source: PLoS One. 2024 Nov 18;19(11):e0311232. doi: 10.1371/journal.pone.0311232 (PMC11573215; doi:10.1371/journal.pone.0311232)
Supplement: S4 Fig — (PDF) [file pone.0311232.s004.pdf]

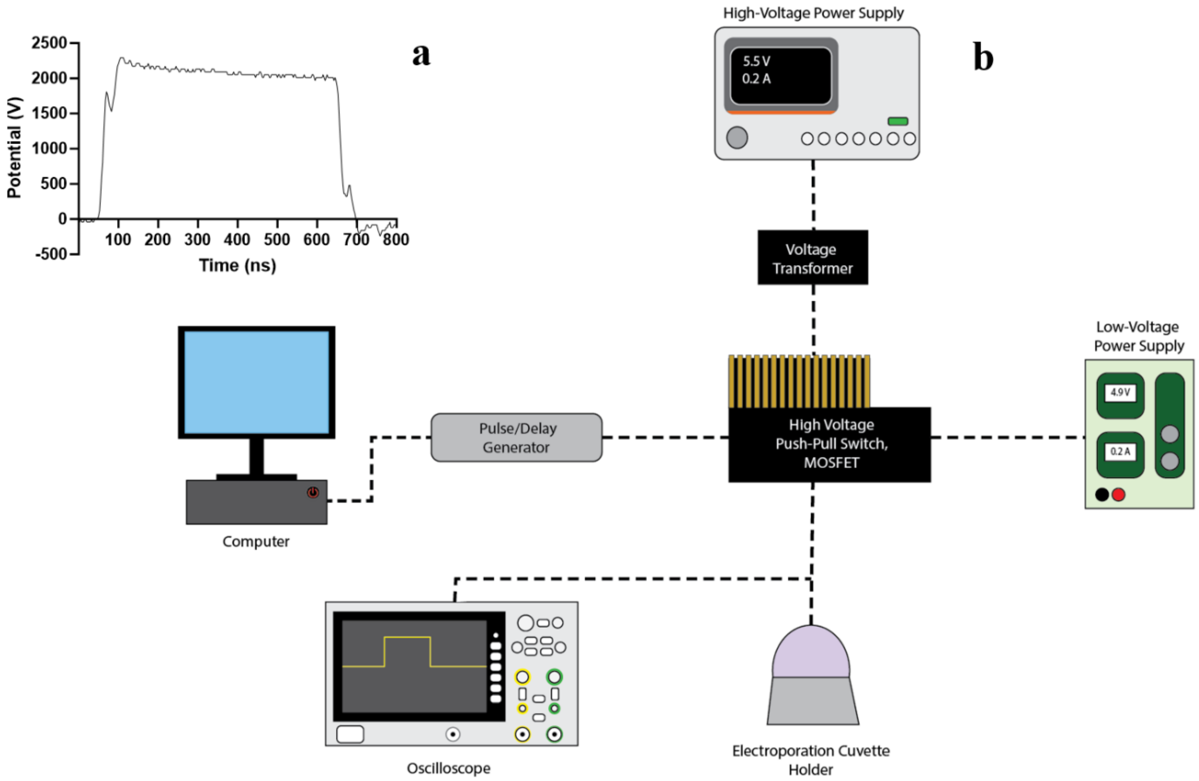

Figure S4. Pulse shape produced from the custom electroporator (a) and diagram of the main components that make up the pulser (b).
