# Supplementary figures and images for: Inactivation of bacteria using synergistic hydrogen peroxide with split-dose nanosecond pulsed electric field exposures
Source: PLoS One. 2024 Nov 18;19(11):e0311232. doi: 10.1371/journal.pone.0311232 (PMC11573215; doi:10.1371/journal.pone.0311232)

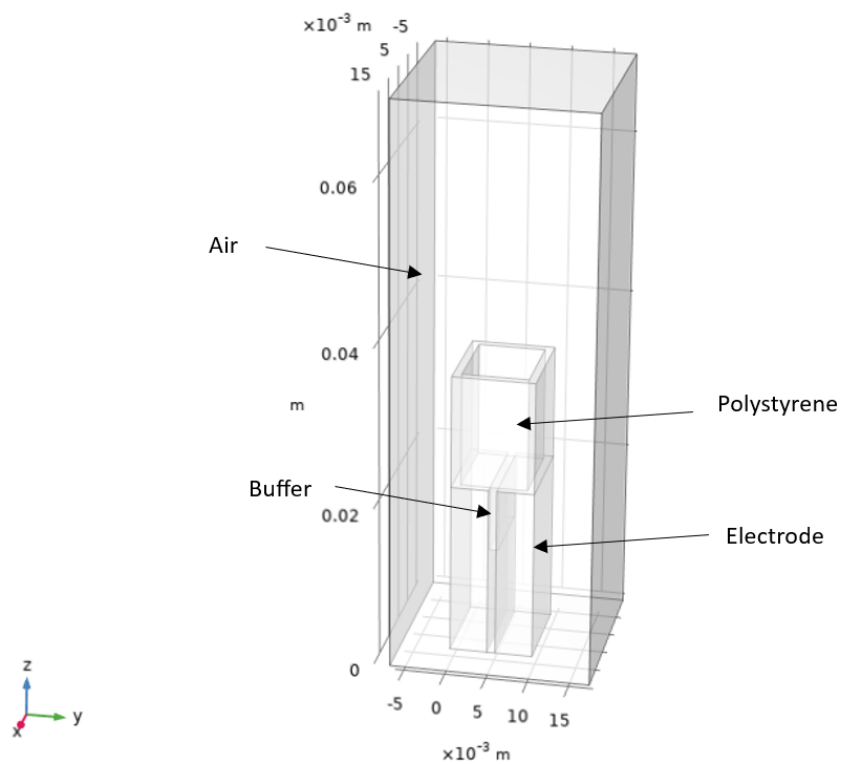

**Figure S5.** Labeled image of electroporation cuvette modeled in COMSOL.

Supplement: S5 Fig — (PDF) [file pone.0311232.s005.pdf]
