# Supplementary material for: Inactivation of bacteria using synergistic hydrogen peroxide with split-dose nanosecond pulsed electric field exposures
Source: PLoS One. 2024 Nov 18;19(11):e0311232. doi: 10.1371/journal.pone.0311232 (PMC11573215; doi:10.1371/journal.pone.0311232)
Supplement: S6 Fig — (PDF) [file pone.0311232.s006.pdf]

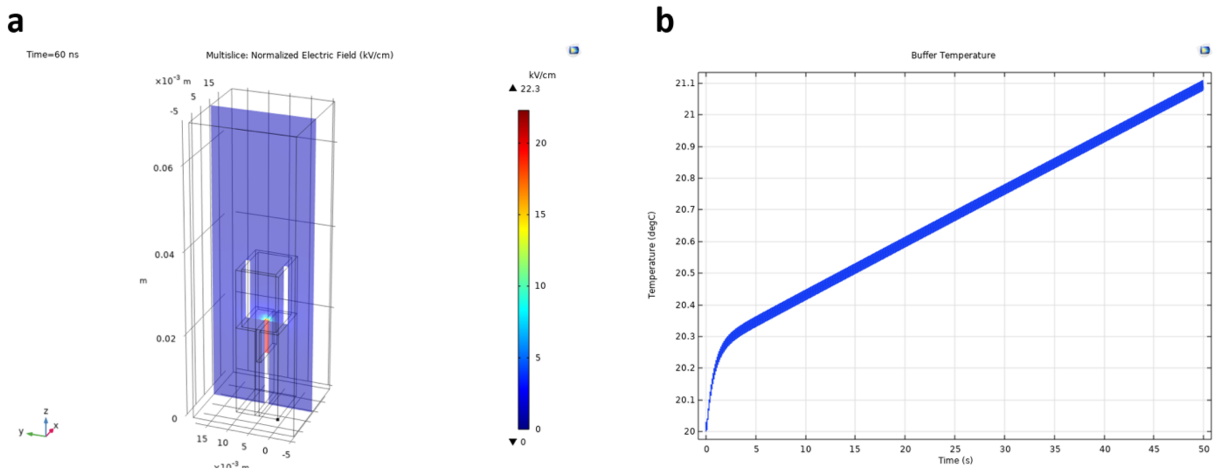

Figure S6. Electric field distribution during a pulse (a) and temperature rise after 500 pulses of 600 ns duration at 21 kV/cm and 10 Hz (b) from COMSOL simulation.
