# Supplementary material for: Inactivation of bacteria using synergistic hydrogen peroxide with split-dose nanosecond pulsed electric field exposures
Source: PLoS One. 2024 Nov 18;19(11):e0311232. doi: 10.1371/journal.pone.0311232 (PMC11573215; doi:10.1371/journal.pone.0311232)
Supplement: S7 Fig — (PDF) [file pone.0311232.s007.pdf]

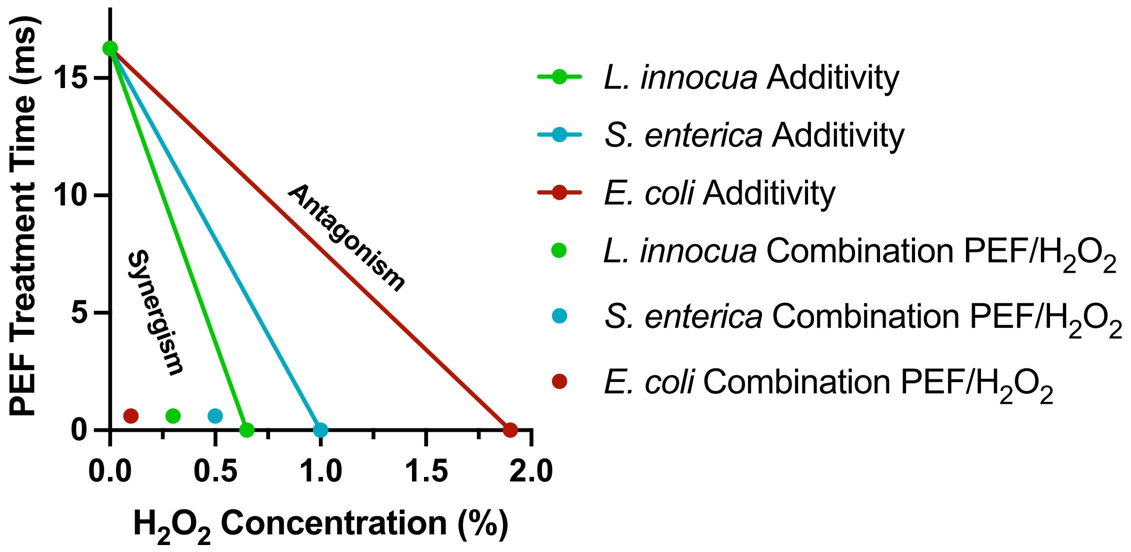

Figure S7. Isobologram of combined PEF/H_2O_2 treatment on different bacterial species describing the necessary conditions to achieve a 5-log reduction in viable population. Combination treatment conditions for each bacterial species leading to this level of disinfection are shown as individual data points. The line of additivity is determined for each species based on the concentration of H_2O_2 and estimation of the PEF time required to reach rapid kill temperature due to Joule heating (see discussion in text). The PEF conditions employed in this study do not even remotely approach this level of intensity. For each bacterial species, combination treatments falling below the line of additivity represent synergy.
