# Supplementary material for: Inactivation of bacteria using synergistic hydrogen peroxide with split-dose nanosecond pulsed electric field exposures
Source: PLoS One. 2024 Nov 18;19(11):e0311232. doi: 10.1371/journal.pone.0311232 (PMC11573215; doi:10.1371/journal.pone.0311232)
Supplement: S1 Table — (PDF) [file pone.0311232.s008.pdf]

Table S1. Experimental evaluation of temperature changes during PEF. For each measurement the initial and final temperature of a solution of Gomori buffer is described. The average temperature increase was found to be +1.8±0.5 ºC.

Entry	Initial Temperature (ºC)	Final Temperature (ºC)	Temperature Increase (ºC)
1	26.5	28.0	1.5
2	25.0	27.5	2.5
3	24.5	26.0	1.5
4	22.0	23.0	1.0
5	24.0	26.0	2.0
6	25.5	27.5	2.0
