# Supplementary material for: Inactivation of bacteria using synergistic hydrogen peroxide with split-dose nanosecond pulsed electric field exposures
Source: PLoS One. 2024 Nov 18;19(11):e0311232. doi: 10.1371/journal.pone.0311232 (PMC11573215; doi:10.1371/journal.pone.0311232)
Supplement: S2 Table — (PDF) [file pone.0311232.s009.pdf]

Table S2. Summary results of statistical analysis of data from Table 1. For each concentration of H_2O_2 evaluated the impact of treatment time and PEF was evaluated.

E. coli 0.1% H2O2

Two-way ANOVA	Ordinary				
Alpha	0.05				
Source of Variation	% of total variation	P value	P value summary	Significant?	
Interaction	8.622	<0.0001	****	Yes	
Row Factor (Treatment Time)	13.01	<0.0001	****	Yes	
Column Factor (PEF)	75.81	<0.0001	****	Yes	
ANOVA table	SS	DF	MS	F (DFn, DFd)	P value
Interaction	7.974	4	1.994	F (4, 18) = 15.16	P<0.0001
Row Factor (Treatment Time)	12.03	2	6.017	F (2, 18) = 45.77	P<0.0001
Column Factor (PEF)	70.11	2	35.05	F (2, 18) = 266.6	P<0.0001
Residual	2.367	18	0.1315		
Tukey's multiple comparisons test	Mean Diff.	95.00% CI of diff.	Below threshold?	Summary	Adjusted P Value
5 min					
0P vs. 1000P	-2.39	-3.146 to -1.634	Yes	****	<0.0001
0P vs. 500+500P	-2.55	-3.306 to -1.794	Yes	****	<0.0001
1000P vs. 500+500P	-0.16	-0.9156 to 0.5956	No	ns	0.8526
10 min					
0P vs. 1000P	-2.793	-3.549 to -2.038	Yes	****	<0.0001
0P vs. 500+500P	-3.193	-3.949 to -2.438	Yes	****	<0.0001
1000P vs. 500+500P	-0.4	-1.156 to 0.3556	No	ns	0.3865
15 min					
0P vs. 1000P	-3.47	-4.226 to -2.714	Yes	****	<0.0001
0P vs. 500+500P	-5.583	-6.339 to -4.828	Yes	****	<0.0001
1000P vs. 500+500P	-2.113	-2.869 to -1.358	Yes	****	<0.0001

E. coli 0.3% H2O2

Two-way ANOVA	Ordinary				
Alpha	0.05				
Source of Variation	% of total variation	P value	P value summary	Significant?	
Interaction	10.5	<0.0001	****	Yes	
Row Factor (Treatment Time)	25.09	<0.0001	****	Yes	
Column Factor (PEF)	60.65	<0.0001	****	Yes	
ANOVA table	SS	DF	MS	F (DFn, DFd)	P value
Interaction	12.49	4	3.122	F (4, 18) = 12.59	P<0.0001
Row Factor (Treatment Time)	29.84	2	14.92	F (2, 18) = 60.15	P<0.0001
Column Factor (PEF)	72.12	2	36.06	F (2, 18) = 145.4	P<0.0001
Residual	4.465	18	0.248		
Tukey's multiple comparisons test	Mean Diff.	95.00% CI of diff.	Below threshold?	Summary	Adjusted P Value
5 min					
0P vs. 1000P	-1.933	-2.971 to -0.8955	Yes	***	0.0004
0P vs. 500+500P	-2	-3.038 to -0.9622	Yes	***	0.0003
1000P vs. 500+500P	-0.06667	-1.104 to 0.9711	No	ns	0.9853

10 min					
0P vs. 1000P	-2.32	-3.358 to -1.282	Yes	****	<0.0001
0P vs. 500+500P	-4.497	-5.534 to -3.459	Yes	****	<0.0001
1000P vs. 500+500P	-2.177	-3.214 to -1.139	Yes	***	0.0001
15 min					
0P vs. 1000P	-4.76	-5.798 to -3.722	Yes	****	<0.0001
0P vs. 500+500P	-4.883	-5.921 to -3.846	Yes	****	<0.0001
1000P vs. 500+500P	-0.1233	-1.161 to 0.9145	No	ns	0.9507

E. coli 0.5% H2O2

Two-way ANOVA	Ordinary				
Alpha	0.05				
Source of Variation	% of total variation	P value	P value summary	Significant?	
Interaction	4.399	0.0011	**	Yes	
Row Factor (Treatment Time)	56.63	<0.0001	****	Yes	
Column Factor (PEF)	36.25	<0.0001	****	Yes	
ANOVA table	SS	DF	MS	F (DFn, DFd)	P value
Interaction	4.515	4	1.129	F (4, 18) = 7.277	P=0.0011
Row Factor (Treatment Time)	58.13	2	29.06	F (2, 18) = 187.4	P<0.0001
Column Factor (PEF)	37.21	2	18.61	F (2, 18) = 120.0	P<0.0001
Residual	2.792	18	0.1551		
Tukey's multiple comparisons test	Mean Diff.	95.00% CI of diff.	Below threshold?	Summary	Adjusted P Value
5 min					
0P vs. 1000P	-1.147	-1.967 to -0.3259	Yes	**	0.0059
0P vs. 500+500P	-1.497	-2.317 to -0.6759	Yes	***	0.0006
1000P vs. 500+500P	-0.35	-1.171 to 0.4707	No	ns	0.5332
10 min					
0P vs. 1000P	-2.513	-3.334 to -1.693	Yes	****	<0.0001
0P vs. 500+500P	-3.07	-3.891 to -2.249	Yes	****	<0.0001
1000P vs. 500+500P	-0.5567	-1.377 to 0.2641	No	ns	0.2212
15 min					
0P vs. 1000P	-3.317	-4.137 to -2.496	Yes	****	<0.0001
0P vs. 500+500P	-3.317	-4.137 to -2.496	Yes	****	<0.0001
1000P vs. 500+500P	0	-0.8207 to 0.8207	No	ns	>0.9999

Listeria 0.1% H2O2

Two-way ANOVA	Ordinary				
Alpha	0.05				
Source of Variation	% of total variation	P value	P value summary	Significant?	
Interaction	24.77	<0.0001	****	Yes	
Row Factor (Treatment Time)	22.68	<0.0001	****	Yes	
Column Factor (PEF)	50.8	<0.0001	****	Yes	
ANOVA table	SS	DF	MS	F (DFn, DFd)	P value
Interaction	2.611	8	0.3264	F (8, 30) = 53.26	P<0.0001
Row Factor (Treatment Time)	2.391	4	0.5977	F (4, 30) = 97.52	P<0.0001
Column Factor (PEF)	5.355	2	2.677	F (2, 30) = 436.8	P<0.0001
Residual	0.1839	30	0.006129		

Tukey's multiple comparisons test	Mean Diff.	95.00% CI of diff.	Below threshold?	Summary	Adjusted P Value
5 min					
0P vs. 1000P	-0.06	-0.2176 to 0.09758	No	ns	0.6205
0P vs. 500+500P	-0.1133	-0.2709 to 0.04425	No	ns	0.1958
1000P vs. 500+500P	-0.05333	-0.2109 to 0.1042	No	ns	0.6849
10 min					
0P vs. 1000P	-0.1367	-0.2942 to 0.02092	No	ns	0.0991
0P vs. 500+500P	-0.5	-0.6576 to -0.3424	Yes	****	<0.0001
1000P vs. 500+500P	-0.3633	-0.5209 to -0.2058	Yes	****	<0.0001
15 min					
0P vs. 1000P	-0.1067	-0.2642 to 0.05092	No	ns	0.2336
0P vs. 500+500P	-0.52	-0.6776 to -0.3624	Yes	****	<0.0001
1000P vs. 500+500P	-0.4133	-0.5709 to -0.2558	Yes	****	<0.0001
30 min					
0P vs. 1000P	-0.66	-0.8176 to -0.5024	Yes	****	<0.0001
0P vs. 500+500P	-1.473	-1.631 to -1.316	Yes	****	<0.0001
1000P vs. 500+500P	-0.8133	-0.9709 to -0.6558	Yes	****	<0.0001
45 min					
0P vs. 1000P	-0.77	-0.9276 to -0.6124	Yes	****	<0.0001
0P vs. 500+500P	-1.597	-1.754 to -1.439	Yes	****	<0.0001
1000P vs. 500+500P	-0.8267	-0.9842 to -0.6691	Yes	****	<0.0001

Listeria 0.3% H2O2

Two-way ANOVA	Ordinary
Alpha	0.05

Source of Variation	% of total variation	P value	P value summary	Significant?
Interaction	24.49	<0.0001	****	Yes
Row Factor (Treatment Time)	44.6	<0.0001	****	Yes
Column Factor (PEF)	28.99	<0.0001	****	Yes

ANOVA table	SS	DF	MS	F (DFn, DFd)	P value
Interaction	39.44	8	4.929	F (8, 30) = 47.72	P<0.0001
Row Factor (Treatment Time)	71.82	4	17.96	F (4, 30) = 173.8	P<0.0001
Column Factor (PEF)	46.68	2	23.34	F (2, 30) = 226.0	P<0.0001
Residual	3.099	30	0.1033		

Tukey's multiple comparisons test	Mean Diff.	95.00% CI of diff.	Below threshold?	Summary	Adjusted P Value
5 min					
0P vs. 1000P	-0.16	-0.8069 to 0.4869	No	ns	0.816
0P vs. 500+500P	-0.27	-0.9169 to 0.3769	No	ns	0.5648
1000P vs. 500+500P	-0.11	-0.7569 to 0.5369	No	ns	0.908
10 min					
0P vs. 1000P	-0.3967	-1.044 to 0.2503	No	ns	0.2999
0P vs. 500+500P	-1.417	-2.064 to -0.7697	Yes	****	<0.0001
1000P vs. 500+500P	-1.02	-1.667 to -0.3731	Yes	**	0.0015
15 min					
0P vs. 1000P	-0.4633	-1.110 to 0.1836	No	ns	0.1983
0P vs. 500+500P	-1.63	-2.277 to -0.9831	Yes	****	<0.0001
1000P vs. 500+500P	-1.167	-1.814 to -0.5197	Yes	***	0.0003

30 min					
0P vs. 1000P	-2.843	-3.490 to -2.196	Yes	****	<0.0001
0P vs. 500+500P	-2.96	-3.607 to -2.313	Yes	****	<0.0001
1000P vs. 500+500P	-0.1167	-0.7636 to 0.5303	No	ns	0.8972
45 min					
0P vs. 1000P	-5.627	-6.274 to -4.980	Yes	****	<0.0001
0P vs. 500+500P	-5.48	-6.127 to -4.833	Yes	****	<0.0001
1000P vs. 500+500P	0.1467	-0.5003 to 0.7936	No	ns	0.8427

Listeria 0.5% H2O2

Two-way ANOVA	Ordinary
Alpha	0.05

Source of Variation	% of total variation	P value	P value summary	Significant?
Interaction	22.73	<0.0001	****	Yes
Row Factor (Treatment Time)	43.13	<0.0001	****	Yes
Column Factor (PEF)	33.73	<0.0001	****	Yes

ANOVA table	SS	DF	MS	F (DFn, DFd)	P value
Interaction	66.32	8	8.29	F (8, 30) = 208.2	P<0.0001
Row Factor (Treatment Time)	125.8	4	31.46	F (4, 30) = 790.3	P<0.0001
Column Factor (PEF)	98.4	2	49.2	F (2, 30) = 1236	P<0.0001
Residual	1.194	30	0.03981		

Tukey's multiple comparisons test	Mean Diff.	95.00% CI of diff.	Below threshold?	Summary	Adjusted P Value
5 min					
0P vs. 1000P	-0.2267	-0.6283 to 0.1750	No	ns	0.3581
0P vs. 500+500P	-0.3	-0.7016 to 0.1016	No	ns	0.1736
1000P vs. 500+500P	-0.07333	-0.4750 to 0.3283	No	ns	0.8947
10 min					
0P vs. 1000P	-0.38	-0.7816 to 0.02163	No	ns	0.0665
0P vs. 500+500P	-1.973	-2.375 to -1.572	Yes	****	<0.0001
1000P vs. 500+500P	-1.593	-1.995 to -1.192	Yes	****	<0.0001
15 min					
0P vs. 1000P	-1.147	-1.548 to -0.7450	Yes	****	<0.0001
0P vs. 500+500P	-2.717	-3.118 to -2.315	Yes	****	<0.0001
1000P vs. 500+500P	-1.57	-1.972 to -1.168	Yes	****	<0.0001
30 min					
0P vs. 1000P	-6.157	-6.558 to -5.755	Yes	****	<0.0001
0P vs. 500+500P	-5.823	-6.225 to -5.422	Yes	****	<0.0001
1000P vs. 500+500P	0.3333	-0.06829 to 0.7350	No	ns	0.1187
45 min					
0P vs. 1000P	-6.12	-6.522 to -5.718	Yes	****	<0.0001
0P vs. 500+500P	-6.12	-6.522 to -5.718	Yes	****	<0.0001
1000P vs. 500+500P	0	-0.4016 to 0.4016	No	ns	>0.9999

Salmonella 0.1%H2O2

Two-way ANOVA	Ordinary
Alpha	0.05

Source of Variation	% of total variation	P value	P value summary	Significant?
Interaction	21.17	<0.0001	****	Yes

Row Factor (Treatment Time)	14.87	<0.0001	****	Yes	
Column Factor (PEF)	59.05	<0.0001	****	Yes	

ANOVA table	SS	DF	MS	F (DFn, DFd)	P value
Interaction	4.58	8	0.5724	F (8, 30) = 16.17	P<0.0001
Row Factor (Treatment Time)	3.216	4	0.8039	F (4, 30) = 22.70	P<0.0001
Column Factor (PEF)	12.77	2	6.387	F (2, 30) = 180.4	P<0.0001
Residual	1.062	30	0.03541		

Tukey's multiple comparisons test	Mean Diff.	95.00% CI of diff.	Below threshold?	Summary	Adjusted P Value
5 min					
0P vs. 1000P	-0.37	-0.7488 to 0.008782	No	ns	0.0566
0P vs. 500+500P	-0.32	-0.6988 to 0.05878	No	ns	0.1105
1000P vs. 500+500P	0.05	-0.3288 to 0.4288	No	ns	0.9434
10 min					
0P vs. 1000P	-0.3567	-0.7354 to 0.02211	No	ns	0.0681
0P vs. 500+500P	-0.7533	-1.132 to -0.3746	Yes	****	<0.0001
1000P vs. 500+500P	-0.3967	-0.7754 to -0.01789	Yes	*	0.0386
15 min					
0P vs. 1000P	-0.4533	-0.8321 to -0.07455	Yes	*	0.0163
0P vs. 500+500P	-1.16	-1.539 to -0.7812	Yes	****	<0.0001
1000P vs. 500+500P	-0.7067	-1.085 to -0.3279	Yes	***	0.0002
30 min					
0P vs. 1000P	-0.5167	-0.8954 to -0.1379	Yes	**	0.0058
0P vs. 500+500P	-1.957	-2.335 to -1.578	Yes	****	<0.0001
1000P vs. 500+500P	-1.44	-1.819 to -1.061	Yes	****	<0.0001
45 min					
0P vs. 1000P	-0.7567	-1.135 to -0.3779	Yes	****	<0.0001
0P vs. 500+500P	-2.273	-2.652 to -1.895	Yes	****	<0.0001
1000P vs. 500+500P	-1.517	-1.895 to -1.138	Yes	****	<0.0001

Salmonella 0.3%H2O2

Two-way ANOVA	Ordinary
Alpha	0.05

Source of Variation	% of total variation	P value	P value summary	Significant?
Interaction	26.1	<0.0001	****	Yes
Row Factor (Treatment Time)	35.28	<0.0001	****	Yes
Column Factor (PEF)	37.41	<0.0001	****	Yes

ANOVA table	SS	DF	MS	F (DFn, DFd)	P value
Interaction	26.53	8	3.316	F (8, 30) = 80.79	P<0.0001
Row Factor (Treatment Time)	35.86	4	8.965	F (4, 30) = 218.4	P<0.0001
Column Factor (PEF)	38.03	2	19.01	F (2, 30) = 463.3	P<0.0001
Residual	1.231	30	0.04104		

Tukey's multiple comparisons test	Mean Diff.	95.00% CI of diff.	Below threshold?	Summary	Adjusted P Value
5 min					
0P vs. 1000P	-0.14	-0.5478 to 0.2678	No	ns	0.6776
0P vs. 500+500P	-0.33	-0.7378 to 0.07779	No	ns	0.1309

1000P vs. 500+500P	-0.19	-0.5978 to 0.2178	No	ns	0.4924
10 min					
0P vs. 1000P	-0.4733	-0.8811 to -0.06555	Yes	*	0.0202
0P vs. 500+500P	-1.617	-2.024 to -1.209	Yes	****	<0.0001
1000P vs. 500+500P	-1.143	-1.551 to -0.7355	Yes	****	<0.0001
15 min					
0P vs. 1000P	-0.9067	-1.314 to -0.4989	Yes	****	<0.0001
0P vs. 500+500P	-2.49	-2.898 to -2.082	Yes	****	<0.0001
1000P vs. 500+500P	-1.583	-1.991 to -1.176	Yes	****	<0.0001
30 min					
0P vs. 1000P	-2.247	-2.654 to -1.839	Yes	****	<0.0001
0P vs. 500+500P	-2.773	-3.181 to -2.366	Yes	****	<0.0001
1000P vs. 500+500P	-0.5267	-0.9345 to -0.1189	Yes	**	0.0092
45 min					
0P vs. 1000P	-4.907	-5.314 to -4.499	Yes	****	<0.0001
0P vs. 500+500P	-3.343	-3.751 to -2.936	Yes	****	<0.0001
1000P vs. 500+500P	1.563	1.156 to 1.971	Yes	****	<0.0001

Salmonella 0.5%H2O2

Two-way ANOVA Alpha	Ordinary 0.05				
Source of Variation	% of total variation	P value	P value summary	Significant?	
Interaction	15.46	<0.0001	****	Yes	
Row Factor (Treatment Time)	40.84	<0.0001	****	Yes	
Column Factor (PEF)	41.55	<0.0001	****	Yes	
ANOVA table	SS	DF	MS	F (DFn, DFd)	P value
Interaction	34.81	8	4.351	F (8, 30) = 26.83	P<0.0001
Row Factor (Treatment Time)	91.96	4	22.99	F (4, 30) = 141.8	P<0.0001
Column Factor (PEF)	93.55	2	46.78	F (2, 30) = 288.5	P<0.0001
Residual	4.865	30	0.1622		
Tukey's multiple comparisons test	Mean Diff.	95.00% CI of diff.	Below threshold?	Summary	Adjusted P Value
5 min					
0P vs. 1000P	-0.4333	-1.244 to 0.3772	No	ns	0.3965
0P vs. 500+500P	-0.43	-1.241 to 0.3806	No	ns	0.402
1000P vs. 500+500P	0.003333	-0.8072 to 0.8139	No	ns	>0.9999
10 min					
0P vs. 1000P	-1.88	-2.691 to -1.069	Yes	****	<0.0001
0P vs. 500+500P	-1.877	-2.687 to -1.066	Yes	****	<0.0001
1000P vs. 500+500P	0.003333	-0.8072 to 0.8139	No	ns	>0.9999
15 min					
0P vs. 1000P	-3.033	-3.844 to -2.223	Yes	****	<0.0001
0P vs. 500+500P	-2.633	-3.444 to -1.823	Yes	****	<0.0001
1000P vs. 500+500P	0.4	-0.4106 to 1.211	No	ns	0.4529
30 min					
0P vs. 1000P	-5.85	-6.661 to -5.039	Yes	****	<0.0001
0P vs. 500+500P	-4.497	-5.307 to -3.686	Yes	****	<0.0001
1000P vs. 500+500P	1.353	0.5428 to 2.164	Yes	***	0.0008
45 min					
0P vs. 1000P	-4.827	-5.637 to -4.016	Yes	****	<0.0001
0P vs. 500+500P	-5.003	-5.814 to -4.193	Yes	****	<0.0001
1000P vs. 500+500P	-0.1767	-0.9872 to 0.6339	No	ns	0.8536
