# Supplementary material for: Inactivation of bacteria using synergistic hydrogen peroxide with split-dose nanosecond pulsed electric field exposures
Source: PLoS One. 2024 Nov 18;19(11):e0311232. doi: 10.1371/journal.pone.0311232 (PMC11573215; doi:10.1371/journal.pone.0311232)
Supplement: Attachment — Submitted filename: Response to Reviewers.docx [file pone.0311232.s010.docx]

**Journal Requirements:**

**When submitting your revision, we need you to address these additional requirements.**

**1. Please ensure that your manuscript meets PLOS ONE's style requirements, including those for file naming. The PLOS ONE style templates can be found at**

[**https://journals.plos.org/plosone/s/file?id=wjVg/PLOSOne_formatting_sample_main_body.pdf**](https://journals.plos.org/plosone/s/file?id=wjVg/PLOSOne_formatting_sample_main_body.pdf) **and** 
[**https://journals.plos.org/plosone/s/file?id=ba62/PLOSOne_formatting_sample_title_authors_affiliations.pdf**](https://journals.plos.org/plosone/s/file?id=ba62/PLOSOne_formatting_sample_title_authors_affiliations.pdf)

**2. We note that the grant information you provided in the ‘Funding Information’ and ‘Financial Disclosure’ sections do not match.**

**When you resubmit, please ensure that you provide the correct grant numbers for the awards you received for your study in the ‘Funding Information’ section.** Sorry for this oversight. The information below is accurate.

**3. Thank you for stating the following financial disclosure:**

**"This work was funded by the USDA National Institute of Food and Agriculture's Agriculture and Food Research Initiative (NIFA AFRI) Food Safety and Defense Program (grant # 2022-67018-36540)."** This information is correct. **Please state what role the funders took in the study.  If the funders had no role, please state: "The funders had no role in study design, data collection and analysis, decision to publish, or preparation of the manuscript." If this statement is not correct you must amend it as needed.** The funders had no role in study design, data collection and analysis, decision to publish, or preparation of the manuscript. This statement has been included in the cover letter as requested.

**Please include this amended Role of Funder statement in your cover letter; we will change the online submission form on your behalf.**

**4. Please note that funding information should not appear in the Acknowledgments section or other areas of your manuscript. We will only publish funding information present in the Funding Statement section of the online submission form. Please remove any funding-related text from the manuscript.** Completed.

**5. Please include captions for your Supporting Information files at the end of your manuscript, and update any in-text citations to match accordingly. Please see our Supporting Information guidelines for more information:** [**http://journals.plos.org/plosone/s/supporting-information**](http://journals.plos.org/plosone/s/supporting-information)**.** This section has been added to the manuscript.

**Additional Editor Comments:**

 I recommend that the article be accepted **after major revisions**. The study presents valuable findings on the synergistic effects of PEF and H2O2 in bacterial inactivation, which is a significant contribution to the field of food safety. However, several areas require improvement to enhance the clarity, depth, and rigor of the manuscript.

**Specific Recommendations for Revision:**

1. **Introduction:**
   - Clearly state the hypothesis of the study to guide the reader's understanding of the research objectives. This has been stated explicitly at the end of our introduction.
2. **Methodology:**
   - Include a flowchart or schematic diagram summarizing the experimental procedures. This has been included in methods and materials section.
   - Provide more justification for the choice of H2O2 concentrations and contact times. This has been included in results section.
3. **Results:**
   - Expand on the statistical analysis, particularly the interpretation of ANOVA results. Additional discussion included. Additional graphical representations of statistical analysis included in Figure 2, Figure 1 moved from Supplement to include graphical representation of statistical analysis in the main document. Full summary output for all statistical analysis ANOVA and multiple comparison tests has been included in the Supplement as Table S2.
   - Discuss the variability in the results, especially concerning the standard deviations, to assess the reliability of the findings. Additional discussion included.
4. **Discussion:**
   - Critically evaluate the limitations of the study, including potential challenges in industrial applications. Additional discussion included.
   - Compare the findings with other recent studies to position this research within the broader field. Additional discussion included.
5. **Presentation:**
   - Proofread the manuscript to correct minor grammatical and typographical errors. The manuscript is believed to now be free of grammatical and typographical errors.

By addressing these revisions and the revisions by the reviewers, the manuscript will be significantly strengthened and will make a substantial contribution to the literature on microbial control and food safety. Thank you. We have made revisions throughout the manuscript. Details of these changes are provided in our point by point response to reviewer comments provided below.

Note: HTML markup is below. Please do not edit.]

Reviewers' comments:

Reviewer's Responses to Questions

**Comments to the Author**

1. Is the manuscript technically sound, and do the data support the conclusions?

The manuscript must describe a technically sound piece of scientific research with data that supports the conclusions. Experiments must have been conducted rigorously, with appropriate controls, replication, and sample sizes. The conclusions must be drawn appropriately based on the data presented.

Reviewer #1: No

Reviewer #2: Yes

Reviewer #3: Yes

Reviewer #4: No

Reviewer #5: Yes

2. Has the statistical analysis been performed appropriately and rigorously?

Reviewer #1: No

Reviewer #2: I Don't Know

Reviewer #3: Yes

Reviewer #4: No

Reviewer #5: Yes

3. Have the authors made all data underlying the findings in their manuscript fully available?

The [PLOS Data policy](https://nam11.safelinks.protection.outlook.com/?url=http%3A%2F%2Fc05y1x9s.r.us-east-2.awstrack.me%2FL0%2Fhttp%3A%252F%252Fwww.plosone.org%252Fstatic%252Fpolicies.action%2523sharing%2F1%2F010f0191479ed82c-f4611e69-fd11-4509-8f6d-ba676ac1def1-000000%2FybKglIb5XvWz3zVcEvbCgsd2mtI%3D171&data=05%7C02%7Cperezla%40studentsrowan.mail.onmicrosoft.com%7Cc5767363dacb4322481d08dcbaf3ace0%7Ceda8e9bc72cf449ca4e6725e6c6bd0d8%7C0%7C0%7C638590805087430381%7CUnknown%7CTWFpbGZsb3d8eyJWIjoiMC4wLjAwMDAiLCJQIjoiV2luMzIiLCJBTiI6Ik1haWwiLCJXVCI6Mn0%3D%7C0%7C%7C%7C&sdata=%2BekVtrIMSxPQcHatFZoxHsHfiKqW%2FK08iBx4%2FdH5ZL0%3D&reserved=0) requires authors to make all data underlying the findings described in their manuscript fully available without restriction, with rare exception (please refer to the Data Availability Statement in the manuscript PDF file). The data should be provided as part of the manuscript or its supporting information, or deposited to a public repository. For example, in addition to summary statistics, the data points behind means, medians and variance measures should be available. If there are restrictions on publicly sharing data—e.g. participant privacy or use of data from a third party—those must be specified.

Reviewer #1: Yes

Reviewer #2: Yes

Reviewer #3: Yes

Reviewer #4: Yes

Reviewer #5: Yes

4. Is the manuscript presented in an intelligible fashion and written in standard English?

PLOS ONE does not copyedit accepted manuscripts, so the language in submitted articles must be clear, correct, and unambiguous. Any typographical or grammatical errors should be corrected at revision, so please note any specific errors here.

Reviewer #1: No

Reviewer #2: Yes

Reviewer #3: Yes

Reviewer #4: No

Reviewer #5: Yes

5. Review Comments to the Author

Please use the space provided to explain your answers to the questions above. You may also include additional comments for the author, including concerns about dual publication, research ethics, or publication ethics. (Please upload your review as an attachment if it exceeds 20,000 characters)

**Reviewer #1:** The paper reports on the combination of PEF and hydrogen peroxide to inactivate Listeria, Salmonella and E. coli in Gomori buffer. The research area is of interest although there are concerns over the structure of the script, experimental approach and interpretation of results. The Abstract could be made more informative to include the methodology and key results. The Introduction should provide more details on the state-of-the-art and basis for the study. Indeed, it was only when reading the Discussion that the terms of split-dose was actually defined. The Materials and Methods should be described in sufficient detail to allow replication. Also there is a need to describe what constitutes treatment time. From the text it appears the holding period when the hydrogen peroxide was added.
The Discussion is primarily composed of speculative statement that have no data to support the various theories. Indeed, the study reads as preliminary and observational with little attention for elucidation of mechanisms. In addition, it would have been informative to have performed trials in a food system given this was highlighted several times within the script.
Specific points
Title: Which bacteria? Which matrix? Clarified in the manuscript as requested.
Abstract
Line 5: In what matrix? Clarified in manuscript as requested.
Line 6: What are split-dose trains? Was PEF applied for 5-45 mins? Traditional PEF exposures involve one train of pulses. In our case, we deliver 1000 pulses of 600 ns duration. Split-dose exposures have been primarily investigated for mammalian cell inactivation, but induces the electrosensitization effect. By splitting the single train in half (2 deliveries of 500 pulses in our case) with a delay in between, it has been seen that even though the same amount of total energy is being delivered to the system, cells are more susceptible to the second train and inactivation increases. We investigate this with bacteria. The 5-45 mins only refers to contact time with hydrogen peroxide. This has been clarified by stating the number of pulses that are delivered in the abstract.
Line 9: What is the contact time? PEF is typically a brief exposure. See above comment.
Line 11: What were the log reductions for the individual treatments? This data is included in Table 1 (H2O2 only, zero “0” P PEF column and Supplemental Figure S1 for PEF only) and is graphically displayed in Figure 2 (Figure 1 from the original submission).
Introduction
Line 5: There are biological options. Thank you. Wording has been changed to emphasize chemical and physical methods are more common, but not the only options.
Line 9: What about irradiation and thermal sterilization? Irradiation is promising but is currently limited in several countries for the purposes and foods it can be applied to. Fresh produce can only be treated for pest control in Australia ([https://www.foodstandards.gov.au/consumer/foodtech/irradiationd](https://www.foodstandards.gov.au/consumer/foodtech/irradiation)) and much of the EU is limited to herbs and spices ([https://agrinfo.eu/book-of-reports/food-irradiation-explained/tform](https://agrinfo.eu/book-of-reports/food-irradiation-explained/)) for example. Thermal sterilization, while it can effectively kill microbes, can not be used for produce that is intended to be consumed raw due to adverse changes in quality. Our introduction presents the use of “non-ionizing radiation” and provides references for this technology. We have additionally clarified the introduction to be more focused on “non-thermal” methods.
Line 12: Do the authors mean non-thermal methods to retain the raw quality of foods? Yes. Thank you. This is now clarified in the manuscript.
Line 17: Is heat generated? This depends on many factors. In our study we observe temperature changes of approximately 2^o^C during the course of PEF. A discussion is given further into the manuscript.
Line 22: Norovirus is the number one cause of foodborne illness. Of the bacteria, Salmonella, Campylobacter and Clostridium perfringens are the most common. E. coli and Listeria are the most virulent. Thank you. We fully agree Norovirus is the most common cause of foodborne illness. In terms of bacterial outbreaks in the U.S., *E. coli*, *Listeria*, and *Salmonella* are the three main causes (see ref 18 & 19). This is clarified in the manuscript.
Line 27: Sub-lethal treatments essentially means negligible log count reductions. The authors need to expand on the benefits of nsPEF and how these differ to normal PEF in terms of pulse time & waveform. Sublethal means less then lethal. For our study nsPEF by itself (individual treatment) does show “essentially negligible” log count reductions (~1 log CFU reduction, see Figure 2 and Supplemental Figure S1). Additional benefits of nsPEF are described (lower temperature increases, lower energy input per pulse, limited electrolysis). Typical pulse durations (μs-ms) are now listed in the manuscript.
Line 14 and throughout: Statements should be supported by citing references. References have been added as requested.
Line 23: Unclear what is meant by split-dose. Clarified in manuscript as requested.
Line 31: The authors should expand on previous work on synergistic effects. Which bacteria, which matrix and how was synergistic action demonstrated? Synergistic action has been expanded upon, as requested including lead references for interested readers.
Line 42: Low concentrations of hydrogen peroxide followed by an antioxidant dip are typically applied. The authors should elaborate on maximum levels and allowable residue levels. Thank you. Clarified in manuscript as requested.
Line 44: There are codex standards for agricultural water. Thank you. This is now reflected in the manuscript.
Line 4: Hydrogen peroxide is relatively expensive compared to other sanitizers. It can also result in bleaching of foods. While many scholars describe hydrogen peroxide as “low cost,” we understand this can be subjective, and it has been removed from the manuscript. Bleaching has also been added as an additional potential effect.
Line 10: What was the rationale for selecting the bacteria to test? Which matrix? The bacteria selected are the three largest contributors to outbreaks among bacteria in the United States. The matrix is now reflected in the manuscript.
Results
Table 1: Should include statistical analysis. Statistical analysis now included in Figure 1. Full statistical analysis of all data reported in Figure 1 has been included as Supplemental Table S1.
Line 18: Assume 3000P refers to 3999 pulses. Disinfection of what? No, 3000P refers to 3,000 pulses not 3999 pulses. “Disinfection” is changed to “inactivation.”
Line 21: Species or genera? Thank you. Genera. This is changed in the manuscript.
Line 24 and throughout: The authors should compare data in statistical terms. Statistical analysis is now shown in Figure 1. P values have been added where deemed appropriate in the manuscript. Full statistical analysis report is also included as Supplemental Table S1.
Line 4: The paragraph starting “effectiveness of PEF” is confusing and unclear what point the authors are trying to make. It is unclear what single or split-dose infers. Clarified in manuscript as requested.
Line 20: What log reductions are obtained? Is a 5 log CFU reduction required? Log reductions are recorded in Table 1. A 5 log reduction (99.999% inactivation of bacteria) is a general FDA rule required for food decontamination. This is the goal that was set for this project as representing an effective treatment.
Line 18: Increased time between pulses? This should be clarified in the revised script. Clarified in manuscript as requested.
Discussion
Line 10-28: This belongs in the Introduction. The authors need to discuss the results obtained in the current study. While we agree that this paragraph does provide background information and could be included in an expanded introduction we believe that it is less relevant as “introduction” and more relevant in context of the following paragraph in the discussion. Accordingly, we respectfully request to leave this paragraph in the discussion portion of the manuscript.
Line 1: Was the work published? This should be described in the Introduction. Yes. This work is cited in text and is also included in the introduction.
Line 6: Could the hydrogen peroxide sensitize the cells to PEF rather than vice versa? Yes. This is also discussed as a possible mechanism of action. For example, from the abstract: “Split-dose PEF exposures enhance lethality for several tested conditions, indicating greater susceptibility to PEF after oxidative damage has occurred.”
Line 8: The authors should avoid speculating on mechanisms given no studies, apart from SEM, was performed. Light microscopy was performed to rule out clumping. The bactericidal mechanism of hydrogen peroxide is well known. The mechanism behind electrosensitization, despite some studies regarding it, is still mostly speculative at this point. Additionally we note that even if the mechanism of inactivation of both H2O2 and PEF were fully known the individual treatments are NOT effective and the combination treatment may therefore have a fully unique mechanism of action. We have carefully evaluated plausible mechanistic processes that may be involved and wish to convey these in our discussion as potentially valuable ideas for future investigations.
Line 20: It is more likely the split dose causes inversions in the membrane potential, although it depends on the waveform. We are not aware of studies showing split-dose PEF causing inversions in bacterial membranes. This study was performed with monopolar PEF pulse sequences, we do not use bipolar PEF pulse sequences which we would agree would possibly lead to inversions in membrane potential for both split dose and single dose PEF treatments. We have no evidence to believe inversions in membrane potential are relevant in the current study.
Line 22: Catalase would degrade hydrogen peroxide rather than hydroxyl-radicals. Agreed. This is clarified in the manuscript
Line 16: Was the temperature monitored? We predicted minimal temperature increase of approximately 2^o^C using a COMSOL model. We did not monitor temperature during our studies. We have repeated our validation of the COMSOL model with selected samples by collecting experimental data that is now included in the supporting information.
Line 20: What plant cells specifically? Clarified in the manuscript as requested.
Line 32: Speculative on applications. References have been added to further clarify and support wastewater applications.
Figure 5 can be removed as it doesn’t contribute to the work Isobologram analysis is a well-established and validated method to demonstrate synergy between two treatments. We believe that rigorously demonstrating synergy is one of the key contributions from this work. Figure 5 has been moved to the Supplemental file, as requested.
Materials and Methods
Line 7: Bacteria and cultivation conditions is a better title. Rewritten in the manuscript as requested.
Line 8: What was the rationale for selecting the bacteria? How were the bacteria stored and revived? Rationale is given in the introduction and storage information is now included.
Line 12: How was agitation applied? Clarified in manuscript as requested.
Line 16: What is the composition of Gomori buffer? Clarified in manuscript as requested.
Line 22: What did an OD 0.1 equate to in CFU/ml? Clarified in manuscript as requested.
Line 23: How confident were the researchers that the hydrogen peroxide was not degraded by the cells prior to PEF treatment. It is possible that a small % of hydrogen peroxide had been degraded, but timing was carefully controlled so that all treatments can be compared accurately relative to one another. PEF exposures were done soon after H2O2 addition to limit degradation.
Line 28: How was the hydrogen peroxide neutralized prior to enumerating survivors? As described in the methods section, hydrogen peroxide was not neutralized prior to enumerating survivors. Hydrogen peroxide at the concentrations employed in this study is sublethal to the bacteria evaluated. See, for example, treatments of bacteria with 0P PEF in Table 1.
Line 20: How long did it take to deliver 1000 pulses? Clarified in manuscript as requested.
Line 34: The authors applied an unusual method for plating by spotting followed by tipping the plate. How were the bacteria evenly distributed using the method? Spotting samples followed by tipping the plate produces very straight, even lines down the length of the plate. The “track-dilution” method provides CFU enumeration equivalent to CFU enumeration using other protocols for this analysis however maintains the benefit of considerably lower environmental impact and waste production. Leading references for this method are provided in the manuscript for interested readers. This method is well established in the field being used by hundreds of publications including multiple publications in this journal.

**Reviewer #2:** The manuscript "Inactivation of Bacteria Using Synergistic Hydrogen Peroxide with Split-Dose Nanosecond Pulsed Electric Field Exposures" is generally well-written and presents results that are well and extensively discussed. However, there are some aspects of the scientific language that should be addressed, as well as the presentation of results.
Page 3, line 2: “The decontamination of microbial contamination…”. There is redundancy and inaccuracy in this sentence. In fact, the decontamination is of the product itself and usually results in a decrease in the contamination…;
This error is repeated throughout the manuscript, for example on page 4 (line 27), page 5 (line 18), page 6 (lines 3 and 7), page 10 (line 29); These have been rewritten in the manuscript.
Page 3, line 27: “...the inactivation of other microbes like viruses…”. Viruses are biological agents but not microbes; Rewritten in manuscript.
Page 4, lines 4-7: the sentence should be rewritten for the sake of clarity; Clarified in manuscript as requested.
Figure 1: the bars in Figure 1, specifically those for treatment with hydrogen peroxide alone, do not match the values presented in Table 1; The data in the Table 1 does indeed match the data presented in Figure 1. Figure 1 has been modified to present different time points and/or H2O2 concentrations based on the comment below.
Page 8, line 4: “The effectiveness of PEF treatments varies between the different bacteria, however, is impacted in a dose-dependent manner correlated to contact time for all bacterial species evaluated”. According to Table 1, this is not true for Salmonella (single train 0.1% 5 min and 10 min, 0.5%, 30 and 45 min); Yes, indeed there are exceptions to this trend. We have modified Figure 1 to better demonstrate the “general trend” in our data while maintaining the result from Salmonella in which this trend is indeed not perfectly maintained. Log reductions generally increase with increasing contact time/concentration, with a few exceptions.
Page 17, lines 1-3: The author claims that temperature shifts after PEF were determined using the COMSOL software. Authors should present the initial and final real temperatures (not estimated) after the several PEF treatments; Yes, we do use the COMSOL software for predictions of temperature changes in PEF as described in the methods and materials. To provide additional support for the limited effect of temperature (~2^o^C temperature change) under the treatment conditions used in this study we have experimentally determined the temperature increase. These additional experimental results are included in the supplement. We would further note that our control experiments in which bacteria are treated with PEF only show very limited inactivation. The predicted (and now experimentally determined) temperature changes of ~2^o^C during PEF treatment is expected to have no impact whatsoever on bacterial viability.

Page 13, line 39 to page 14, line 10: authors acknowledge that heating caused by electric pulses has an effect on cell death and use the COMSOL software to calculate the temperature increase. This is a fundamental issue, and the authors should clarify whether they measured temperatures before and after the PEF treatment. Additionally, it would be interesting to understand the effect of thermal treatment on the reduction of microbial load at the temperature reached after PEF, but without the use of PEF. Temperatures were not recorded before and after each PEF treatment and this has now been clarified in the manuscript. The accuracy of the COMSOL model has been validated. We include additional experimental data in supporting information in support of this. The “thermal effect” from the treatment from a ~2^o^C temperature change as we described in the manuscript would have NO impact on the reduction of bacterial load. We don’t believe it would be “interesting to understand the effect of thermal effect” as we have already clearly demonstrated in this manuscript that PEF alone (resulting in the same “thermal effect” suggested by this reviewer) does NOT result in effective inactivation of bacteria.

The description on Page 13, line 39 to page 14, line 10 was referring to a THEORETICAL calculation of the required pulses to bring the solution to an instant kill temperature and this is NOT representative of the conditions used in this study. This figure is now removed to avoid confusion and per the request of another reviewer.

The temperature change from either 500 or 1,000 pulses as employed in the study is minimal and has very little effect on the treatment. Especially in regard to *L. innocua* and *S. enterica*, PEF is rather ineffective on its own (Figure 3) so temperature equivalents would show little to now reductions in microbial load. While the authors agree temperature equivalents are useful in some studies, it would not provide much useful information in this one.

**Reviewer #3:** The authors did a good job in this manuscript. They combined nsPEF with H2O2 to kill different strains of bacteria. The results showed an enhanced effect for the combination compared to each technique separate. The introduction showed clearly the advantages of using nsPEF over micro or millisecond PEF in saving power. Description of the experimental procedures was concise and contained detailed descriptions of well-established procedures. The results fully discussed.

We thank this reviewer for their comments and positive evaluation of out manuscript.

**Reviewer #4:** The manuscript presents an interesting study on the use of Pulsed Electric Fields (PEF) combined with hydrogen peroxide for microbial inactivation. While the concept has potential applications in food safety, it would be more relevant to test this method on a food matrix rather than in a buffer system. The methodology needs clearer explanation and correction in certain sections. Additionally, the results are limited and not sufficient for a scientific publication, and the discussion is largely speculative. Overall, the manuscript, in its current form, lacks sufficient data and rigorous testing to fully support its conclusions. Further details and suggestions are provided below.

Title: What bacteria were tested? Title should be more descriptive. Clarified in the manuscript as requested.

Abstract
Line 4: Was the bacterial reduction achieved in water, food matrix, or another medium? Clarified in the manuscript as requested.
Line 8: CFU per? Clarified in the manuscript as requested.
Line 8: Which condition resulted in more than 5 log reductions? Clarified in the manuscript as requested.
Line 10: ‘’Split-dose PEF exposures enhance lethality for several tested conditions, indicating greater susceptibility to PEF after oxidative damage has occurred.’’ How and why is its effect different from single dose? Do you have any evidence for your conclusions? Greater log reductions are shown in several of the tested conditions. This effect is a general trend however and is not observed in every situation.

Key words: The selected keywords do not accurately reflect the focus of the study.

Introduction
The introduction begins by discussing food decontamination, yet the method described in the paper is not applied to food. This discrepancy should be addressed for clarity. Although the research gap is identified, it would benefit from a clearer and more detailed articulation. The stated objectives could be more specific. For instance, the rationale behind combining PEF and hydrogen peroxide needs to be better explained. It would be helpful to understand the underlying theory and the expected outcomes of using these two methods together. Clarifying these aspects would provide a stronger foundation for the study’s aims and enhance the overall coherence of the paper.
- Page 3, Line 14: "PEF in food processing?" Consider expanding on why PEF is chosen for this study and its significance in food processing. A brief overview of its advantages would be helpful. Clarified in the manuscript as requested.
- Page 4, Line 34: "Hydrogen peroxide role?" Provide a brief explanation of why hydrogen peroxide is used in conjunction with PEF. What is its expected role in microbial inactivation? Clarified in the manuscript as requested.
- Page 5, Line 8: "Herin?" The word "Herin" seems to be a typo. It should be "Herein" or another word depending on the intended meaning. Thank you. Corrected in the manuscript.

Results
Several figures labeled as supplementary are essential for a comprehensive understanding of the results, particularly Figures S1, S2, and S3. These figures are critical for elucidating the process described. However, the paper lacks sufficient data to be considered a rigorous scientific study, and many of the figures largely duplicate the information found in Table 1. Furthermore, although COMSOL modeling is discussed in the methodology section, its outcomes are not presented in the results section. To improve the paper, it is crucial to conclude which treatment combinations were most effective and to offer a brief interpretation of these findings directly within the results section. This would enhance the clarity and depth of the analysis provided.
- Page 5: What were the results for split-dose PEF treatment alone—45 min? The supplementary figures are necessary to give readers an idea of the process. PEF treatment alone is found in the “Electrosensitization” section of the Results (Figure 3).
- Page 6, Line 10: ‘’No bacterial growth observed following treatment.’’ Do you mean enrichment? How do you consider it? What was the limit of detection in your trial? “No bacterial growth observed following treatment” refers to a plate with no colony formation after the incubation period. This is now clarified in the manuscript and the LOD is now stated in M&M.
- Table 1: The format of the table is not suitable for a manuscript. Thank you. We would please request PLoS ONE editor guidance on better format for the table.
- Table 1: In order to compare the data in the table, a statistical analysis is needed. Thank you for the suggestion. Statistical significance has now been included in Figure 1. Additionally all statistical analysis for Table 1 has been included in the Supplement as Table S1.
- Figure 1: What was your reason for choosing these conditions to show in the picture? If you wanted to show the best results for each treatment, for E. coli wasn’t it 0.5%–15 min? If it shows the conditions with lower concentrations of H2O2 that resulted in more than a 5 log CFU/ml reduction of bacteria, for E. coli it is 0.1%, 15 min, split-dose PEF. Thank you. Several conditions could easily have been chosen, as the purpose of this figure was to highlight the synergy between PEF and H2O2 which is observed in many of the treatments. Conditions are now chosen as the lowest H2O2 concentration for each bacteria to reach a 5 log reduction, hopefully enhancing readability.
- Figure 2: It is mentioned in the abstract that ‘’Split-dose PEF exposures enhance lethality for several tested conditions, indicating greater susceptibility to PEF after oxidative damage has occurred.’’ Based on the picture, this ability is only observed in the condition of 0.1% hydrogen peroxide addition to 0.3% H2O2 only for E. coli. What is the reason for better/similar results of a single dose in a higher dosage of hydrogen peroxide? This figure has been removed to improve clarity. The added Figure 1 and Figure 4 better represent this ability. Split-dose treatments are seen to be more effective at all concentrations (aside from 0.5% and Salmonella), but this general trend is dependent on contact time. A few speculations are given for why higher dosages may inhibit efficacy of the second train, but future works will be needed to more completly investigate possible mechanisms of action.
- Page 8, Line 15 to 30: What are the reasons for these results? Contact time or concentration of hydrogen peroxide? We hypothesize it is due to reduced PEF efficiency or the higher initial concentrations of bacteria for *L. innocua* and *S. enterica*. These rationales are given in the discussion.
- Page 9, Line 10: What do you conclude by observing the microscopic pictures? (I wasn’t able to find the supplemental figures). These images are included as Supplemental Figure S3 (previously Figure S4). The absence of bacterial clumping reduced the likelihood of one possible reason for the decreased efficacy of split dose exposures with increasing contact times in H2O2. Based on this observation additional discussion is focused on other possible mechanism of action.
- Page 9, Line 12 to 14: Significance of findings? the statistical analysis is needed for Table 1 to be able to discuss it. The results for single and split doses on Listeria reduction for 0.5% and 0.3% H2O2 for 30 and 45 min look similar. Statistical significance is now presented in Figure 1. In addition, all statistical analysis is included in the Supplemental File as Table S1.
- Page 9, Line 14 to 22: Why did PEF delay time have an effect on bacterial availability? What is the theory behind it? Why is Salmonella more resistant to PEF delay time compared to Listeria and E. coli? This is a great question. There is often a tailing of survival curves with an increasing number of pulses. The electrosensitization theory suggests that by splitting up a train of pulses into two with a delay in between, cells can resensitize to PEF and inactivation can be enhanced. The theory suggests that this is because pores that are formed are conductive and will inhibit a small proportion of the population from further membrane charging. The delay allows cells to reseal the formed pores and once again be susceptible to membrane charging within an electric field. This postulated effect is discussed in the introduction. It is uncertain why Salmonella specifically is more resistant to delay time, however, we suggest it may be due to overall PEF resistance from a higher charging time constant (due to the known larger cell radius of Salmonella compared to E. coli and Listeria). This hypothesis is mentioned in the discussion.

Discussion
The discussion presents various theories and ideas from other researchers, but it lacks organization and comprehensiveness in explaining the synergistic effect between PEF and hydrogen peroxide. For example, while the authors frequently address the formation of hydroxyl radicals, it remains unclear whether these radicals are produced when PEF is combined with hydrogen peroxide. Clarifying this potential formation could significantly enhance the interpretation of the results. We agree and we are hopeful that our publication and discussion will stimulate this and other investigations aimed at better defining the mechanism of action of PEF by itself on cells let alone the mechanism of action for PEF in combination with H2O2. Additionally, the discussion does not provide sufficient evidence from the results to support its speculative claims. We have begun investigations into the mechanism of action for PEF which is incompletely understood currently. The complexity and extent of these studies is well beyond the scope of this manuscript. To strengthen the paper, it would be beneficial to compare the effectiveness of the PEF-hydrogen peroxide combination with other microbial inactivation methods, providing valuable context. Comparison studies to other microbial inactivation methods is difficult. What additional methods are “comparable”? It is unclear what additional value demonstrating that 10% bleach is equally effective to 0.3% H2O2 + PEF would provide for this manuscript. We believe that the exceptional synergy observed between very low concentrations of H2O2 and PEF has significant value as a potential method for bacterial inactivation and our manuscript is focused on relaying this discovery to the scientific community. It is likely that numerous factors and extensive comparative analysis will be required for the development of each specific microbial treatment process. These studies for specific applications are beyond the scope of this manuscript. Furthermore, the paper should address any study limitations and propose directions for future research, especially regarding the scalability of PEF-hydrogen peroxide treatment in industrial applications. Indeed true. Thank you. The limitations of this method have been more completely described in the discussion.
- Page 10, Line 24 to 28: "With a compatible electrode material and a buffer consisting of no added transition metal ions, the H2O2 reduction must be catalyzed by bioavailable ions. It is estimated that a third of all proteins contain metal and 40% of enzymes with known structures are metal-dependent. While most of the total metal content within a bacterium cell is bounded, free metal ions also exist on the μM scale. H2O2 will specifically oxidize enzymes and proteins bonded to iron, in addition to Fe2+ ions within the labile pool [85]. Pore formation from PEF will cause either the efflux of intracellular contents or the influx of H2O2, exposing the chemical to a much higher concentration of metal ions compared with diffusion. The quicker formation of hydroxyl radicals will generate more significant damage to the surrounding proteins, enzymes, lipids, and DNA.’’ Based on this theory, is it better to use PEF and hydrogen peroxide at the same time or expose the bacteria to PEF first and then add hydrogen peroxide? Hydrogen peroxide first, then PEF. This is now reflected in the Discussion.
- Page 11, Line 13, 14: ‘’In a general sense, we hypothesize that oxidative damage to the membrane structure induces greater susceptibility to PEF.’’ How do you hypothesize the damage in the membrane without microscopy consideration? The mechanism of PEF is known to involve changes in membrane structure and potential. While it is possible intracellular contents can be affected by a PEF exposure, the pulse duration needs to be below the membrane charging time constant (the 600 ns pulses used in this study are not) and the field strength needs to be sufficiently high (21 kV/cm is not for bacteria). These predictions are fully in line with our observations that the PEF treatments alone are not effective in bacterial inactivation. Therefore, we postulate that the lethal action of PEF must be membrane-based.
- Page 12, Line 2: Which evidence suggests oxidative damage aids in enhancing susceptibility to PEF exposures? Only increasing the log reduction level? Yes, the extent of inactivation of split-dose treatments compared to single train is amplified with H2O2 vs. without. The ability of H2O2 to cause oxidative damage is known. Therefore, the introduction of a chemical that is capable of causing oxidative damage (although at concentrations at which the oxidative effects of this chemical are sublethal) with PEF does indeed enhance bacterial inactivation.

Materials and Methods
- Page 15, Line 5: "Initial microbial levels?" Mention the initial levels of microorganisms and the detection limits used in the study. This is crucial for interpreting the effectiveness of the treatments. Clarified in the manuscript as requested.
- Page 15, Line 15: "Buffer choice?" Explain the rationale behind the choice of buffer, pH, and the concentration range of hydrogen peroxide. How do these factors influence the results? Clarified in the manuscript as requested.
Page 15, Line 30, a Schematic picture of the unit will help the reader understand the process better Flowchart is added (Figure 5)
Page 16, line 35, is 16 h enough for growing Listeria, E.coli and Salmonella on LB? Yes. Listeria colonies are small after 16 h, although visible. They were incubated for additional time for better clarity in pictures, and this is now reflected in the manuscript.

Page 17, line 24, wasn’t better to use an electron microscope to consider the membrane damage instead of a light microscope? A light microscope allows for a better understanding of how bacteria interact with each other in solution. This was done to rule out clumping as a possible reason for split-dose treatments losing efficacy with longer contact times. SEM studies (similar to those described in reference 121 in the manuscript) may be valuable to assess membrane damage during combination treatment in addition to extensive additional mechanistic evaluations should be incorporated in future studies, which is now reflected in the manuscript.

**Reviewer #5:** The paper aims to fill a gap in the literature for nsEP application as a means of bacterial disinfection. Specifically, the authors note that nsEP alone and nsEP plus antibiotics have been investigated to some success as well as split dose approaches to increase inherent sensitivity of the system to nsEP but neither in combination. They therefore focus on assessing the combined effects of split dose nsEP and hydrogen peroxide. The authors introduce H202 as a optimal means of sterilization across many applications due to its relatively non-toxic nature, disposal via dissociation, low cost, and lack of smell/taste. The authors did well to expose three unique bacteria types to nsep, h202, and combined exposures. Overall, they found that all bacteria were impact by the combined exposure and split exposure impacted all bacteria (although the effect was not universal or straightforward). The authors did a great job of controlling exposure time across all samples and concentrations of h202. This is admirable as I found myself asking questions about consistency in incubation time of the cells per exposure paradigm and found they had noted in the methods that they had held all samples for the same amount of time under each condition. This is rarely done in papers and shows a high level of control and quality of the experiments performed. Additionally, the results and discussion sections are thorough and leave little unturned as possible mechanism(s) for the results or future studies to do. The author does mention that oxidation of phospholipids has been both shown in impact nsEP sensitivity and also be driven by nsEP exposure (in a couple papers). This is a key point as the point of action of H202 in these bacteria is not identified by the author nor its dependence on time well explored. Understandably this is beyond the goal of this paper, but it is key to determining if the mechanism is truly functioning on amplification of the same mechanism or an interplay between two stressed that presents as reduction in bacterial population. The key drawback of the paper is therefore the lack of a mechanism of action outside of speculation in how these two stressors are working together on the bacteria. it is noted that the mechanism for nsEP remains unknown for cells both mammalian and bacteria, so it would be difficult for the authors do anything more than speculate and maintain the focus of this paper on the synergetic effects of nsEP and h020 exposures. We agree and we have initiated investigations to hopefully contribute to this challenging question in the field. We hope that our manuscript stimulates continued investigations in this area. We acknowledge and thank this reviewer for their knowledge of the field and understanding of the complexity associated with determinations of the mechanism of action for nsPEF.

The paper was well written and I have no negative comments regarding its quality. The paper was thoroughly reviewed by the authors and the figures were made clearly and concisely. I do question how applicable nsEP and H202 could be in a commercial application, but given this papers results, you would definitely need less of both to achieve a similar outcome of either alone. This would drive down costs of pulser systems which are currently being deployed in clinical waste water applications. This paper defined a gap in knowledge regarding nsEP for bacterial decontamination and thoroughy filled it beyond expectations by studying a large range of parameters across three dissimilar species. The authors also noted future work that needs to be done to better refine and describe the results. I believe this paper should be accepted for publication. We thank this reviewer for their positive comments and are pleased that they strongly support the manuscript for publication.

Minor Points:
Some of the supplemental figures could easily be in the paper as the trends with increasing dose are better understood by graph as opposed to table, especially unexpected trends.
Page 8 Line 4 “..however is impacted” reads odd. Thank you. Corrected in the manuscript.

6. PLOS authors have the option to publish the peer review history of their article ([what does this mean?](https://nam11.safelinks.protection.outlook.com/?url=https%3A%2F%2Fc05y1x9s.r.us-east-2.awstrack.me%2FL0%2Fhttps%3A%252F%252Fjournals.plos.org%252Fplosone%252Fs%252Feditorial-and-peer-review-process%2523loc-peer-review-history%2F1%2F010f0191479ed82c-f4611e69-fd11-4509-8f6d-ba676ac1def1-000000%2Fa8O0Non5SZG4f9f4PQQ06TQpUB4%3D171&data=05%7C02%7Cperezla%40studentsrowan.mail.onmicrosoft.com%7Cc5767363dacb4322481d08dcbaf3ace0%7Ceda8e9bc72cf449ca4e6725e6c6bd0d8%7C0%7C0%7C638590805087434833%7CUnknown%7CTWFpbGZsb3d8eyJWIjoiMC4wLjAwMDAiLCJQIjoiV2luMzIiLCJBTiI6Ik1haWwiLCJXVCI6Mn0%3D%7C0%7C%7C%7C&sdata=iuqXivUUX3EiAkB%2BcEUcpqjQIE1qx0djXwyVek89nX0%3D&reserved=0)). If published, this will include your full peer review and any attached files.


If you choose “no”, your identity will remain anonymous but your review may still be made public.


**Do you want your identity to be public for this peer review?** For information about this choice, including consent withdrawal, please see our [Privacy Policy](https://nam11.safelinks.protection.outlook.com/?url=https%3A%2F%2Fc05y1x9s.r.us-east-2.awstrack.me%2FL0%2Fhttps%3A%252F%252Fwww.plos.org%252Fprivacy-policy%2F1%2F010f0191479ed82c-f4611e69-fd11-4509-8f6d-ba676ac1def1-000000%2F3IAaL6D3pFo24hNfmUWZz4OhbyI%3D171&data=05%7C02%7Cperezla%40studentsrowan.mail.onmicrosoft.com%7Cc5767363dacb4322481d08dcbaf3ace0%7Ceda8e9bc72cf449ca4e6725e6c6bd0d8%7C0%7C0%7C638590805087439453%7CUnknown%7CTWFpbGZsb3d8eyJWIjoiMC4wLjAwMDAiLCJQIjoiV2luMzIiLCJBTiI6Ik1haWwiLCJXVCI6Mn0%3D%7C0%7C%7C%7C&sdata=F7CqHW5C915oOnSr1AdO1PURtUEOBTj9nuEljuP2Jdo%3D&reserved=0).

Reviewer #1: **Yes:** Keith Warriner

Reviewer #2: No

Reviewer #3: **Yes:** Amr Abd-Elghany

Reviewer #4: No

Reviewer #5: No

[NOTE: If reviewer comments were submitted as an attachment file, they will be attached to this email and accessible via the submission site. Please log into your account, locate the manuscript record, and check for the action link "View Attachments". If this link does not appear, there are no attachment files.]

While revising your submission, please upload your figure files to the Preflight Analysis and Conversion Engine (PACE) digital diagnostic tool, [https://pacev2.apexcovantage.com/](https://nam11.safelinks.protection.outlook.com/?url=https%3A%2F%2Fc05y1x9s.r.us-east-2.awstrack.me%2FL0%2Fhttps%3A%252F%252Fpacev2.apexcovantage.com%252F%2F1%2F010f0191479ed82c-f4611e69-fd11-4509-8f6d-ba676ac1def1-000000%2FJFvS_EOg981qVlVEJKBTn2Aj1E0%3D171&data=05%7C02%7Cperezla%40studentsrowan.mail.onmicrosoft.com%7Cc5767363dacb4322481d08dcbaf3ace0%7Ceda8e9bc72cf449ca4e6725e6c6bd0d8%7C0%7C0%7C638590805087443812%7CUnknown%7CTWFpbGZsb3d8eyJWIjoiMC4wLjAwMDAiLCJQIjoiV2luMzIiLCJBTiI6Ik1haWwiLCJXVCI6Mn0%3D%7C0%7C%7C%7C&sdata=FUk35G0FRxqjLnvvywExdx6si4JWez0ORM9KiWBrAT4%3D&reserved=0). PACE helps ensure that figures meet PLOS requirements. To use PACE, you must first register as a user. Registration is free. Then, login and navigate to the UPLOAD tab, where you will find detailed instructions on how to use the tool. If you encounter any issues or have any questions when using PACE, please email PLOS at [figures@plos.org](mailto:figures@plos.org). Please note that Supporting Information files do not need this step.
